# Overexpression of MusaMYB31, a R2R3 type MYB transcription factor gene indicate its role as a negative regulator of lignin biosynthesis in banana

**DOI:** 10.1371/journal.pone.0172695

**Published:** 2017-02-24

**Authors:** Himanshu Tak, Sanjana Negi, T. R. Ganapathi

**Affiliations:** 1 Plant Cell Culture Technology Section, Nuclear Agriculture and Biotechnology Division, Bhabha Atomic Research Centre, Trombay, Mumbai, India; 2 Homi Bhabha National Institute, Anushakti Nagar, Mumbai, India; Institute of Genetics and Developmental Biology Chinese Academy of Sciences, CHINA

## Abstract

Lignin and polyphenols are important cellular components biosynthesized through phenylpropanoid pathway. Phenylpropanoid pathway in plants is regulated by some important transcription factors including R2R3 MYB transcription factors. In this study, we report the cloning and functional characterization of a banana R2R3-MYB transcription factor (*MusaMYB31*) by overexpression in transgenic banana plants and evaluated its potential role in regulating biosynthesis of lignin and polyphenols. Sequence analysis of MusaMYB31 indicated its clustering with members of subgroup 4 (Sg4) of R2R3MYB family which are well known for their role as repressors of lignin biosynthesis. Expression analysis indicated higher expression of *MusaMYB31* in corm and root tissue, known for presence of highly lignified tissue than other organs of banana. Overexpression of *MusaMYB31* in banana cultivar Rasthali was carried out and four transgenic lines were confirmed by GUS histochemical staining, PCR analysis and Southern blot. Histological and biochemical analysis suggested reduction of cell wall lignin in vascular elements of banana. Transgenic lines showed alteration in transcript levels of general phenylpropanoid pathway genes including lignin biosynthesis pathway genes. Reduction of total polyphenols content in transgenic lines was in line with the observation related to repression of general phenylpropanoid pathway genes. This study suggested the potential role of *MusaMYB31* as repressor of lignin and polyphenols biosynthesis in banana.

## Introduction

Lignin is an important macromolecule with roles in providing mechanical strength to the plant, supporting water transport through xylem tissue and biotic stress tolerance [1]. Lignin is one of the most abundant components of biomass and is synthesized through phenylpropanoid pathway which is responsible for synthesis of secondary metabolites such as polyphenols and flavonoids as well [2,1]. Covalent cross linking of lignin and other aromatic phenolic compounds with cellulose prevent efficient enzymatic digestion of cellulose and thus reducing the proficient conversion of lignocellulosic biomass to bioethanol [3, 4, 5]. Lignin biosynthesis during secondary cell wall development is regulated by transcription factors belonging to *NAC* and R2R3 *MYB* gene families [6]. Members of R2R3 MYB gene family have been clustered into 22 subgroups on the basis of certain domains excluding the highly conserved MYB domain [7]. Members of subgroup 4 (Sg4) have been characterized as repressors of lignin biosynthesis pathway [8, 9]. Members of subgroup 4 (C2 repressor motif clade) shared the presence of conserved C2 repressor core (D/E)LNL(D/N)L associated with EAR (ethylene response factor-associated amphiphilic repression) motif [7, 10, 11]. Multiple reports on MYB transcription factors have emerged over the years indicating their potential roles in regulating the general phenylpropanoid pathway. Two MYB factors of *Antirrhinum majus* namely AmMYB308 and AmMYB330 has been reported as repressors of lignification and phenolic acid metabolism and they were able to down-regulate the expression of *CAD* (cinnamyl alcohol dehydrogenase), *4CL* (4-coumarate:CoA ligase) and *C4H* (cinnamate-4-hydroxylase) in transgenic tobacco plants [12]. *Arabidopsis* MYB4 regulate sinapate esters involved in UV protection as it down-regulates the *C4H* (cinnamate-4-hydroxylase) [10]. Maize, ZmMYB42 down-regulate several genes of lignin and flavonoid biosynthesis pathway and thus reducing lignin content besides qualitatively altering lignin composition [2]. Another maize gene, ZmMYB31 repress many genes involved in the synthesis of monolignols resulting in reduction of lignin content in transgenic plants increasing their cell wall degradability [13]. Four of the grapevine R2R3 MYB proteins (containing C2 repressor core) have been recently characterized as repressors of general phenylpropanoid biosynthetic genes. Overexpression of *VvMYB4a* and *VvMYB4b* limit the synthesis of small weight phenolic compounds while *VvMYBC2-L1* and *VvMYBC2-L3* overexpression resulted in severe reduction in petal anthocyanins and seed proanthocyanidins [14]. Switchgrass (*Panicumvirgatum*) MYB transcription factor, PvMYB4 can down-regulate the expression of monolignol pathway genes by binding to *AC-elements* in their promoter and its overexpression cut down the lignin biosynthesis in transgenic switchgrass [15].

In the present study we have investigated the role of a R2R3 MYB transcription factor belonging to C2 repressor clade from banana and analyzed its potential role in regulating the phenylpropanoid pathway genes after overexpression in transgenic banana plants. Our results suggest that *MusaMYB31* is an efficient repressor of lignin deposition as it down-regulate many genes involved in lignin biosynthesis in transgenic banana plants. Further, transgenic banana plants also display remarkable reduction in total polyphenolic and lignin content along with repression in expression of many genes involved in general phenylpropanoid pathway genes. Results obtained suggest that MusaMYB31 is a functional orthologous of ZmMYB31 which has been reported as a competent repressor of lignin biosynthesis in maize [13]. Present report will augment our understanding about regulation of lignin biosynthesis in banana plants.

## Material and methods

### Source of banana plants

The banana plants used in this study are regenerated in the laboratory from the embryogenic cell suspension (ECS) of *Musa* cultivar Rasthali described below in the section “generation of transgenic banana plants overexpressing *MusaMYB31*”. The control banana plants were maintained on shoot multiplication medium (MS medium with 2 mg/l BA and 30 mg/l adenine sulphate) and rooted on MS medium with 1mg/l NAA.

### RNA isolation and first strand cDNA synthesis

Expression of *MusaMYB31* in different organs of two month old banana (growing in green house) was carried out using real time RT-PCR. Tissue of three uniform plants was mixed in equal amount for RNA isolation. Total RNA isolation from various tissue of banana cultivar Rasthali (leaves, pseudostem, petiole, corm and roots) as well as from embryogenic cells was carried out using Concert plant RNA reagent (Invitrogen, USA) and cleaned by RNA binding column of RNeasy plant mini kit (Qiagen, Germany).First strand cDNA was made using RevertAid first strand cDNA synthesis kit (Thermo Fisher Scientific) as per the manufacturer instruction.

### Expression analysis of *MusaMYB31* by real time RT-PCR

Transcript level analysis of *MusaMYB31* in different organ of banana was carried out by quantitative RT-PCR analysis. Synthesized cDNA was diluted 1:50 with molecular biology grade water and used for QPCR using JumpStar Taq ReadyMix (2X) (Sigma, USA) following the manufacturer instruction. The expression of Banana *EF1α* (reference gene)was used for normalization of different C_t_-values. Expression of *MusaMYB31* in embryogenic cells of banana cultivar Rasthali was used for determination of fold value change in transcript level of *MusaMYB31* in different organs. Readings of experiment was subjected to comparative Ct method (2^−ΔΔCt^) to calculate the fold change of *MusaMYB31*as described previously [16]. Real time RT-PCR for expression of *MusaMYB31* in various tissues was performed at least three times.

### Amplification and cloning of full length *MusaMYB31* coding sequence

Full length coding sequence of *MusaMYB31* was amplified from leaf cDNA of banana cultivar Rasthali. PCR conditions used are: 94°C (5min) followed by 35 cycles of 94°C (20 sec), 56°C (30 sec) and 72°C (50 sec) which was followed by a final extension at 72°C for 5 minutes. Amplified cDNA was PCR purified (High Pure PCR Product Purification Kit; Roche), digested with *Sbf*I and *Kpn*I followed by gel extraction (Roche). The *nos* 3’-UTR was cloned in *Sac*I and *EcoR*I sites of multiple cloning site (MCS) in *pCAMBIA1301* and the recombinant vector (*pCAMBIA1301*-*nos*) was further digested with *Hind*III and *Kpn*I. *Zea mays* polyubiquitin promoter (digested with *Hind*III and *Pst*I) and *MusaMYB31* (digested with *Sbf*I and *Kpn*I) were ligated in a three way ligation with *pCAMBIA1301-nos* (digested with *Hind*III and *Kpn*I) to generate *pCAMBIA1301-MusaMYB31*. The construct was sequenced to confirm the cloning and coding sequence integrity of *MusaMYB31*(amplified by PCR technique). The complete coding sequence of *MusaMYB31* has been deposited in NCBI database with an accession number of KU507534.

### Sequence analysis of MusaMYB31

The theoretical *MusaMYB31*coding sequence translation, pI (isoelectric point) and molecular weight of the MusaMYB31 was predicted using online available tools at expasy server (www.expasy.org). Sequence based similarity searching was performed in NCBI database using pBLAST search. Related R2R3 MYB factors which are annotated and assigned for function are used for building a neighbor joining tree (boot strap replicate of 1000) with the help of clustal omega (http://www.ebi.ac.uk/Tools/msa/clustalo/) and MEGA using default parameters [17]. Conserved motif search in multiple sequence alignment of R2R3 MYB transcription factors was conducted using the MEME software (http://meme-suite.org/) with parameters: 3 to 10 residues, maximum five motifs and zero or one occurrence of motifs.

### Generation of transgenic banana plants overexpressing *MusaMYB31*

Transgenic lines of banana were generated after transformation of embryogenic cell suspension (ECS) of *Musa* cultivar Rasthali as described earlier [18]. *Agrobacterium tumefaciens* strain *EHA105* [19] transformed with *pCAMBIA1301-MusaMYB31* was grown to optical density of 0.5 and was then induced with 100 μM ACS (acetosyringine). Further, the bacterium was co-cultivated with 0.5 ml packed cell volume of ECS of banana cv Rasthali for 30 minutes. Using a vacuum pump and filtration unit, the ECS were then filtered onto a glass fiber filter. The T-DNA transfer events by *Agrobacterium* was allowed for three days in dark after culturing on semi-solid M2-medium [20]. The development of ECS into embryos was carried after culturing the cells on embryo developing medium (BEM) which was supplied with 400mg/l cefotaxime and 5 mg/l hygromycin. Each stage was photographed and the well developed embryos were converted into shoots by culturing on M4 medium (MS medium with 0.5 mg/l BA). Shoot multiplication medium (MS medium with 2 mg/l BA and 30 mg/l adenine sulphate) and rooting medium (MS medium with 1mg/l NAA) were employed for raising in vitro transgenic lines.

### Molecular confirmation of transgenic plants

Leaves of putative transgenic lines were stained with GUS (β-D-Glucuronidase) staining solution using X-Gluc (5-Bromo-4-chloro-3-indolyl Glucuronide) dissolved in sodium phosphate buffer (pH7) containing 1% Triton X-100. Leaf sample was incubated at 37°C overnight and later cleared in 90% methanol. Molecular confirmation of T-DNA insertion events in transgenic banana lines were carried out by PCR and Southern blot followed by transcript level analysis by real time RT-PCR analysis. Genomic DNA of control and transgenic lines isolated using commercially available genomic DNA isolation kit (Sigma, USA; catalogue number G2N350) was analyzed for T-DNA insertion by PCR amplification of *hpt-II* (*hygromycin phosphotransferase*) gene. PCR conditions utilized are 94°C (6 min), 38 cycles of 94°C (20 sec), 52°C (30sec), 72°C (40 sec) and finally 72°C for 3 min. Southern blot analysis was performed as described earlier [21]. Genomic DNA (approx. 20μg) of control and transgenic lines digested with 20 units of *Kpn*I overnight was separated on 1.5% agarose gel. Gel resolved digested DNA was transferred by capillary action onto nylon membrane (Make Amersham, RPN.203N) using 10x SSC buffer. Probe was generated using PCR amplified *hpt-II* and DIG labeling kit (Roche;11585614910). Probe was hybridized to membrane fixed DNA at 42°C (overnight) which was followed by stringency washes carried out at room temperature and 65°C (using 2x SSC and 0.5x SSC with 0.1% SDS respectively) to remove excess probe. Blot was developed using appropriately diluted (1:5000) anti-DIG antibody and chemiluminescent substrate CSPD as per instructions supplied with the kit (Roche, 11585614910). Transcript abundance of *MusaMYB31* in transgenic lines was determined by real time RT-PCR. Total RNA isolation from leaves and cDNA synthesis as described above was performed. Real time PCR was performed following the steps described in the heading “Expression analysis of *MusaMYB31*”.

### Histological analysis of secondary wall

Free hand sections of the petiole of control and transgenic banana lines were prepared. The secondary wall was observed with visualization of lignin by either auto-fluorescence or staining with toluidine blue-O. UV (ultra violet) excitation at 365 nm of the petiole sections in a fluorescent microscope (Eclipse 80i, Nikon) was carried out and auto-fluorescence of the lignin in the secondary wall image was recorded. Petiole sections were stained with 0.1% toluidine blue-O solution (dissolved in phosphate buffer, pH 7) for two minutes followed by rigorous washing in water to remove excess stain. The toluidine blue stained sections were observed and image was recorded in an optical light microscope. The histological observation was taken for at least three uniform plants of each confirmed line.

### Estimation of phenolic content in transgenic lines

Tissue of three uniform plants (two month growth in green house) was mixed in equal amount for extraction of phenolics. Total phenolic content in leaves of control and transgenic lines was estimated using Folin–Ciocalteu (FC) colorimetric assay. Fresh leaves (100mg) of control and transgenic lines extracted in 80% methanol were incubated at 4°C for 3 hours. Supernatant collected after centrifugation was made up to 3ml with distilled water. Further, 500 μl of Folin Ciocalteau reagent (diluted with equal volume of water) and 2 ml of 20% Na_2_CO_3_ was mixed with the supernatant which was subsequently incubated for 20 minutes at 48°C. Readings were observed at 650 nm and data was represented in terms of gallic acid equivalents (mg GAE/g).The experiment was repeated at least three times.

### Biochemical estimation of lignin

Lignin estimation using thioglycolic acid reagent was carried out as described earlier [22]. In brief, 300 mg pseudostem tissue of two month old plants (growing in green house) was extracted in absolute methanol and the resulting pellet was mixed and incubated for twenty minutes each with methanol (2x), NaCl (1M), SDS (1%), milli-Q water (2x), ethanol and 1:1 v/v of chloroform/methanol. The pellet obtained thereafter represent the cell wall, which was further dried in an oven (overnight at 60°C). Lignin-thioglycolic acid complex (LTGA) was generated by treating cell wall (10 mg) with thioglycolic acid (300 μl) as well as 2 M HCl (1.2 ml) followed by heating at 95°C (4 hours). The pellet with LTGA was re-suspended in 0.5M NaOH to extract the LTGA (overnight). This was repeated twice and the two alkali extracts were combined together and further acidified with concentrated HCl (300 μl). The resulting solution was incubated at 4°C (4 hours) and the LTGA was recovered after centrifugation. The brown color pellet re-suspended in 0.5 M NaOH was analyzed at 280 nm. Lignin content estimation was carried out in triplicate after mixing the tissue of at least three uniform plants.

### Real time PCR analysis of genes involved in general phenylpropanoid pathway

Total RNA from leaves tissue of control and transgenic lines(plants with growth of two months in green house) was isolated using method as described above. RevertAid first strand cDNA synthesis kit (Thermo Fisher Scientific) used for synthesis of cDNA utilizing 2μg of total RNA. cDNA was diluted to 1:50 with molecular biology grade water and used for real time quantitative RT-PCR on a Rotor gene-Q platform (Qiagen, Germany) with JumpStar Taq ReadyMix (Sigma, USA) as per vendors instructions. Along with genes of lignin biosynthesis pathways and genes involved in general phenylpropanoid pathway, expression of banana *EF1α* (reference gene)was also monitored. Analysis of reading was performed using comparative Ct method (2^−ΔΔCt^) resulting in estimation of fold change in expression of target gene. Primer sequences utilized in the present study for all the molecular biology work carried out are provided in the S1 Table. Real time RT-PCR for gene expression analysis was performed at least three times.

## Results

### Isolation and sequence analysis of *MusaMYB31*

**Fig 1:**
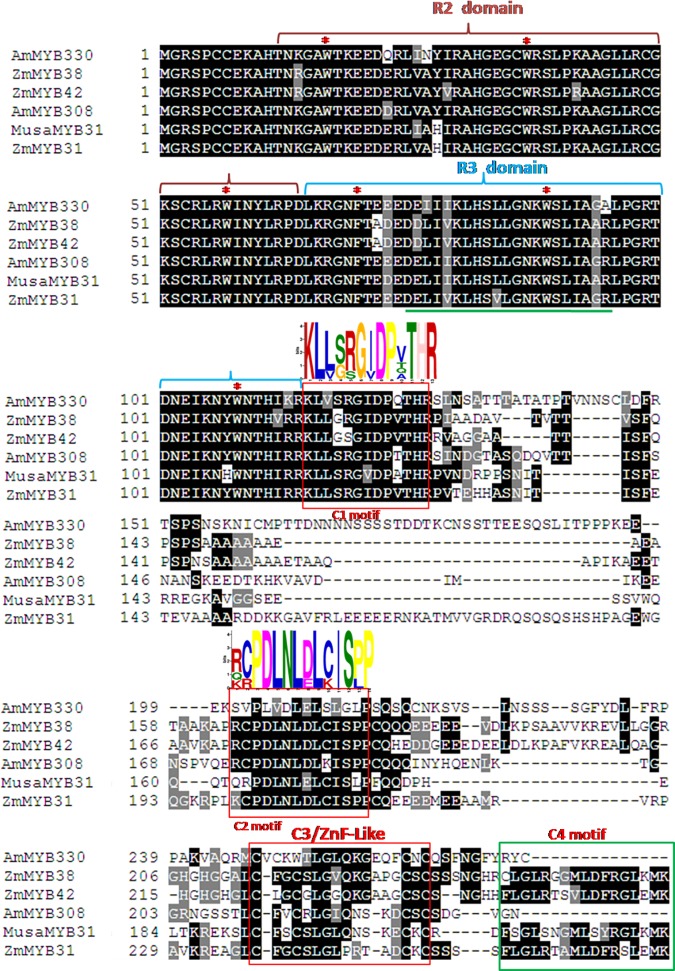
Alignment of MusaMYB31 with other R2R3 MYB repressors belonging to C2 repressor clade. The R2 and R3 domain has been indicated by colored lines on top. The conserved amino acids, tryptophan (W) and phenylalanine (F) are indicated by star. A green line within the R3 domain indicate the motif (consensus sequence [DE]Lx2[RK]x3Lx6Lx3R) interacting with the bHLH protein. The C1 and C2 motifs outside the R2R3 MYB domain are boxed in red. MEME motif logos with bit scores for each position are shown on the top of C1 and C2 motif. The C3/ZnF like and C4 motif are also indicated. Sequences utilized for alignment are: *Antirrhinum majus* AmMYB330 (P81395.1 A), *Zea mays* ZmMYB38 (XP_008664600.1), *Zea mays* ZmMYB42 (NP_001106009.2), *Antirrhinum majus* AmMYB308 (P81393.1) and *Zea mays* ZmMYB31 (NP_001105949.2).

Complete coding sequence of *MusaMYB31* contain 681 bases (NCBI accession number: KU507534) and codes for a protein of 226 amino acids with theoretical pI of 9.4 and molecular weight of 25.8 kDa. *Insilico* analysis for subcellular localization (http://nls-mapper.iab.keio.ac.jp/cgi-bin/NLS_Mapper_form.cgi) indicated the presence of a nuclear localization signal (“RPDLKRGNFTEDEDELIIKLHSL” starting from amino acid at 62^nd^ position) suggesting nuclear localization of MusaMYB31.MusaMYB31 contain the conserved R2R3 domain at the N-terminal end, which is also the DNA binding site of the R2R3 MYB proteins. Characteristic presence of three tryptophan residue (W) in R2 domain and one phenylalanine (F) and two tryptophan residue (W) in R3 domain, important for formation of the core of helix-turn-helix (HTH) [23] were also observed. Alignment of different R2R3 MYB proteins of Sg4 clade with MusaMYB31 also indicated presence of another conserved motif with a consensus sequence [DE]Lx2[RK]x3Lx6Lx3R. This motif within the R3 domain has been suggested to play important role in interaction with the bHLH protein [24, 25] indicating that MusaMYB31 might interact with bHLH protein for their function. MEME software analysis indicated presence of characteristic signature sequences of the Sg4 group [7] of the R2R3 MYB proteins. Apart from C1 and C2 motifs, recently an additional motif known as C3-motif/ZnF-like (zinc finger like) has been identified in the c-terminal of certain repressors belonging to R2R3 MYB transcription factors [14]. Similar C3 motif was also located in the C-terminal of MusaMYB31 suggesting its role as a repressor of general phenylpropanoid pathway (Fig 1).

### Phylogenetic analysis of MusaMYB31

**Fig 2:**
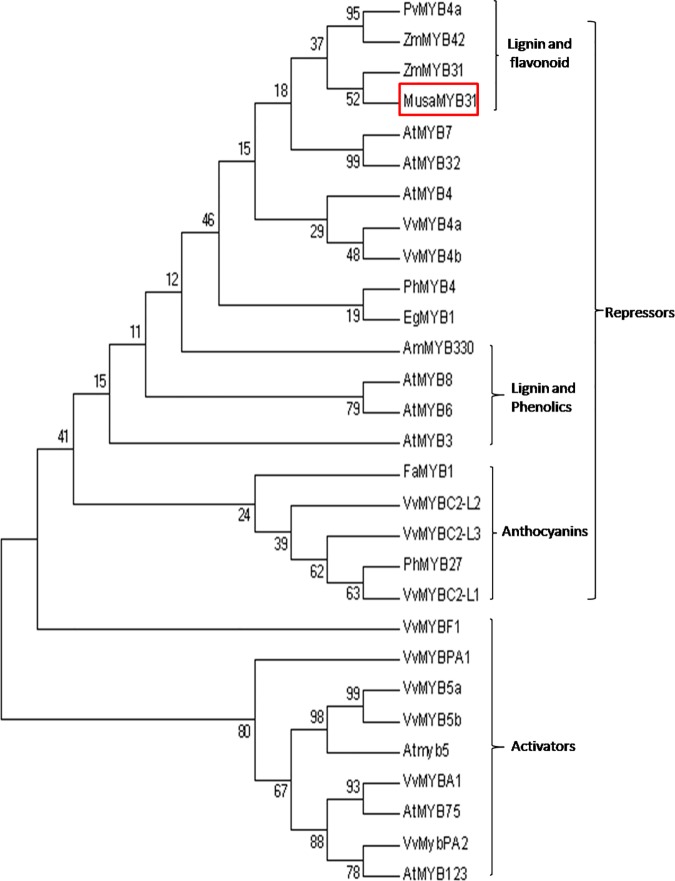
Phylogenetic analysis of MusaMYB31 and other R2R3 MYB transcription factors from different plant species acting as repressors and activators in phenylpropanoid pathway. Predicted and reported function of these MYB factors is indicated. The NCBI accession numbers used in tree construction are: *Panicum virgatum* PvMYB4a (AEM17348.1), *Zea mays* ZmMYB42 (NP_001106009.2), *Zea mays* ZmMYB31 (NP_001105949.2), *Arabidopsis thaliana* AtMYB7 (NP_179263.1), *Arabidopsis thaliana* AtMYB32 (NP_195225.1), *Arabidopsis thaliana* AtMYB4 (AAC83582.1), *Vitis vinifera* VvMYB4a (ABL61515.1), *Vitis vinifera* VvMYB4b (ACN94269.1), *Petunia hybrida* PhMYB4 (ADX33331.1), *Eucalyptus gunnii* EgMYB1 (CAE09058.1), *Antirrhinum majus* AmMYB330 (P81395.1 A), *Arabidopsis thaliana* AtMYB8 (NP_849749.1), AtMYB6 (NP_192684.1), *Arabidopsis thaliana* AtMYB3 (NP_564176.2), *Fragaria ananassa* FaMYB1 (AAK84064.1), *Vitis vinifera* VvMYBC2-L2 (ACX50288.2), *Vitis vinifera* VvMYBC2-L3 (AIP98385.1), *Petunia hybrid* PhMYB27 (AHX24372.1), *Vitis vinifera* VvMYBC2-L1 (ABW34393.1), *Vitis vinifera* VvMYBF1 (ACT88298.1), *Vitis vinifera* VvMYBPA1 (CAJ90831.1), *Vitis vinifera* VvMYB5a (AAS68190.1), *Vitis vinifera* VvMYB5b (Q58QD0), *Arabidopsis thaliana* AtMYB5 (AAC49311.1), *Vitis vinifera* VvMYBA1 (BAD18977.1), *Arabidopsis thaliana* AtMYB75 (AAG42001.1), *Vitis vinifera* VvMYBPA2 (ACK56131.1) and *Arabidopsis thaliana* AtMYB123 (Q9FJA2.1).

As MusaMYB31 sequence analysis suggested presence of multiple motifs commonly found in Sg4 clade of R2R3 MYB transcription factors, hence sequences of multiple R2R3 MYB transcription mostly belonging to Sg4 clade were aligned to build a phylogenetic tree. MusaMYB31 was closely related to maize MYB31 and also show high percentage identity with other R2R3 MYB factors known as repressors and activators of phenylpropanoid pathway genes. MusaMYB31 share identities of 61% with PvMYB4a (AEM17348.1), 55% with ZmMYB42 (NP_001106009.2), 62% with ZmMYB31 (NP_001105949.2), 54% with AtMYB7 (NP_179263.1), 60% with AtMYB32 (NP_195225.1), 57% with AtMYB4 (AAC83582.1), 67% with VvMYB4a (ABL61515.1), 68% with VvMYB4b (ACN94269.1), 62% with PhMYB4 (ADX33331.1), 63% with EgMYB1 (CAE09058.1), 90% with AmMYB330 (P81395.1A), 81% with AtMYB8 (NP_849749.1), 85% with AtMYB6 (NP_192684.1), 55% with AtMYB3 (NP_564176.2), 56% with FaMYB1 (AAK84064.1), 55% with VvMYBC2-L2 (ACX50288.2), 49% with VvMYBC2-L3 (AIP98385.1), 68% with PhMYB27 (AHX24372.1), 56% with VvMYBC2-L1 (ABW34393.1), 56% with VvMYBF1 (ACT88298.1), 69% with VvMYBPA1 (CAJ90831.1), 63% with VvMYB5a (AAS68190.1), 58% with VvMYB5b (Q58QD0), 66% with AtMYB5 (AAC49311.1), 56% with VvMYBA1 (BAD18977.1), 60% with AtMYB75 (AAG42001.1), 66% with VvMYBPA2 (ACK56131.1) and 68% with AtMYB123 (Q9FJA2.1) (Fig 2).

### Transcript level of *MusaMYB31* in different tissues

**Fig 3:**
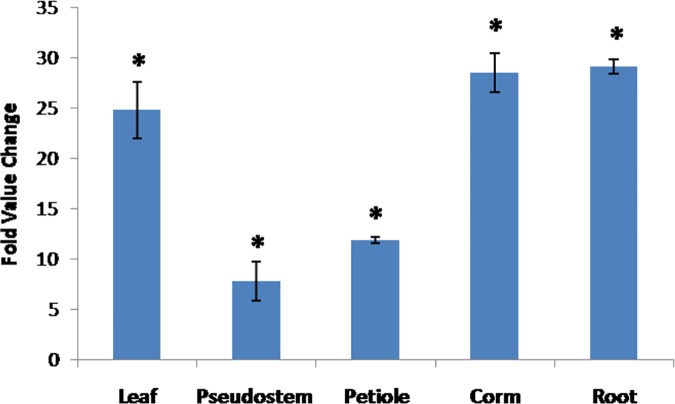
Transcript level analysis of *MusaMYB31* in different organs of the *Musa* cv. Rasthali. Fold value change in expression of *MusaMYB31* was calculated relative to expression in embryogenic cells of banana cv Rasthali. Values are represented as mean± SD. Statistical significant events at 5% are shown with an asterisk (*) on the top.

Using quantitative RT-PCR the transcript abundance of *MusaMYB31* in different organs was analyzed. The expression of *MusaMYB31* in different organs was calculated as fold change relative to expression in embryogenic cells of banana cultivar Rasthali. The lowest expression of *MusaMYB31* was detected in pseudostem followed by petiole. However, very high transcript level was detected in corm and roots followed by leaves. The high level of *MusaMYB31* expression in corm and roots may be due to more lignifying nature of these organs in banana compared to other parts. The difference in expression of *MusaMYB31* in different tissues suggested its possible differential regulation in these tissues (Fig 3).

### Generation of transgenic banana plants overexpressing *MusaMYB31*

**Fig 4:**
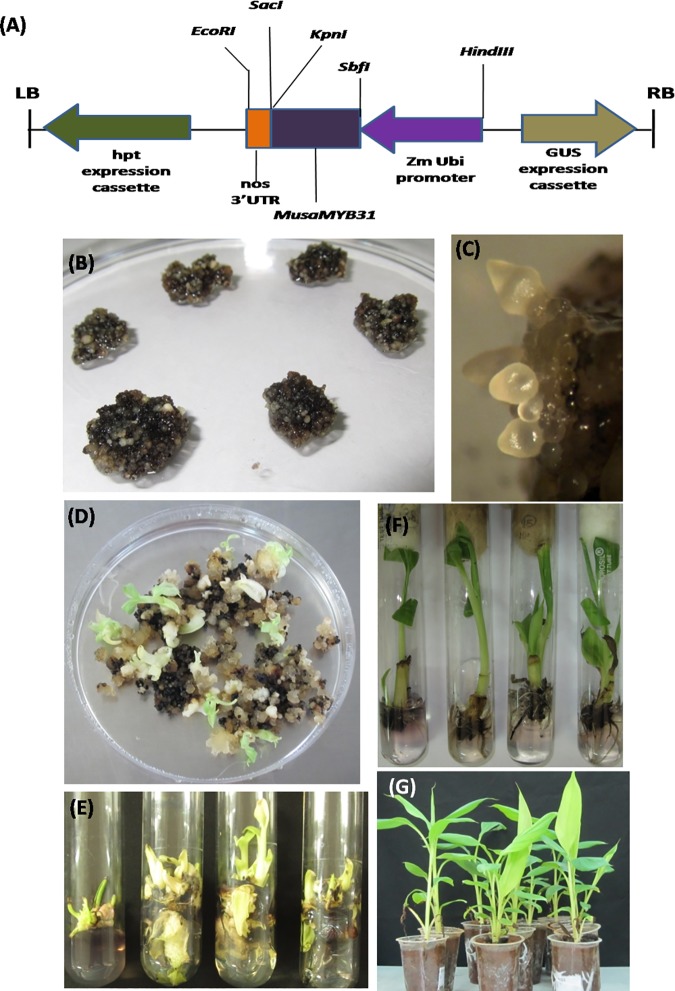
Generation of transgenic banana plants overexpressing *MusaMYB31*. (A)Schematic representation of T-DNA portion utilized to generate transgenic lines. (B) Transformed embryogenic cells of *Musa* cv. Rasthali showing growth on selection medium (BEM with 5 mg/l hygromycin). (C) Close up view of embryos showing development in various stages. (D) Conversion of embryos into shoots on shoot multiplication medium. (E) Generation of multiple shoots of putative transgenic lines. (F) Rooting of different transgenic lines on rooting medium. (G) Hardening of rooted transgenic lines in green house.

The complete coding sequence of *MusaMYB31* was cloned downstream of maize *polyubiquitin* promoter (*pZmUbi*) in *pCAMBIA1301* to generate a cassette for constitutive overexpression of *MusaMYB31* (Fig 4A). Embryogenic cells of banana cultivar Rasthali transformed with the above mentioned construct resulted in emergence of putative embryos on embryo development medium (Fig 4B). The embryos appeared globular and lead to generation of white translucent secondary embryos (Fig 4C). Emerging embryos converted to small shoots on shoot development medium supplemented with hygromycin (5 mg/l) (Fig 4D). Individual shoots were numbered and then multiplied on shoot multiplication medium (Fig 4E). Rooting of putatively transformed shoots was carried out on rooting medium and later hardened in the green house (Fig 4F and 4G).

### Confirmation of transgenic lines for T-DNA integration and their phenotypic analysis

**Fig 5:**
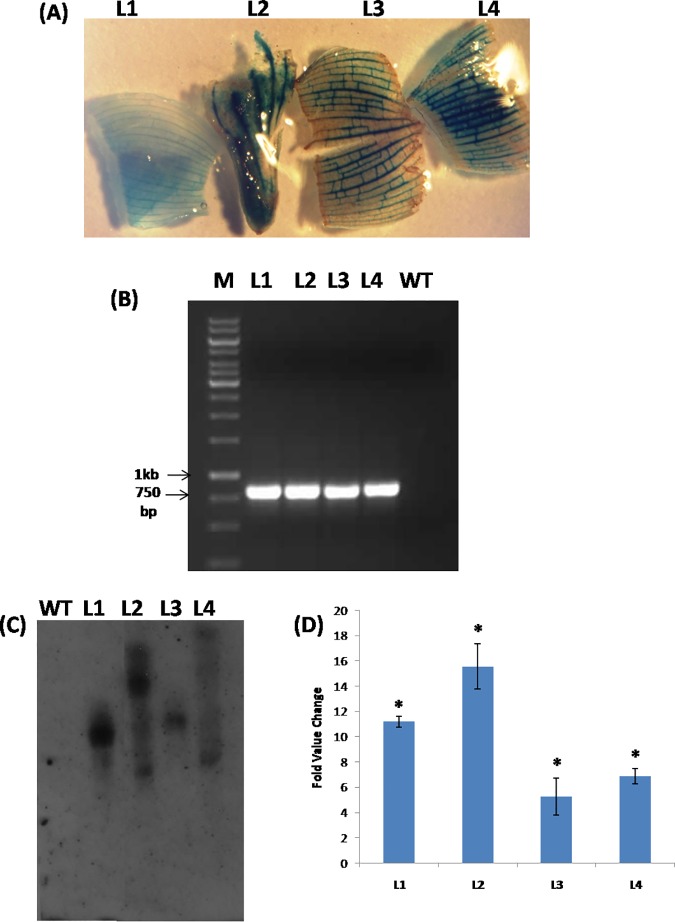
Confirmation of transgenic lines. (A) GUS staining test of putative transgenic lines. (B) PCR analysis of transgenic lines. Amplification of *hpt-II* indicated T-DNA insertion in transgenic lines. (C) Southern blot confirmation of T-DNA insertion in transgenic lines. (D) Real time PCR analysis of *MusaMYB31* transcript level in different transgenic events. Values represented are mean ± SD. Statistical significant events at 5% are shown with an asterisk (*) on the top.

**Fig 6:**
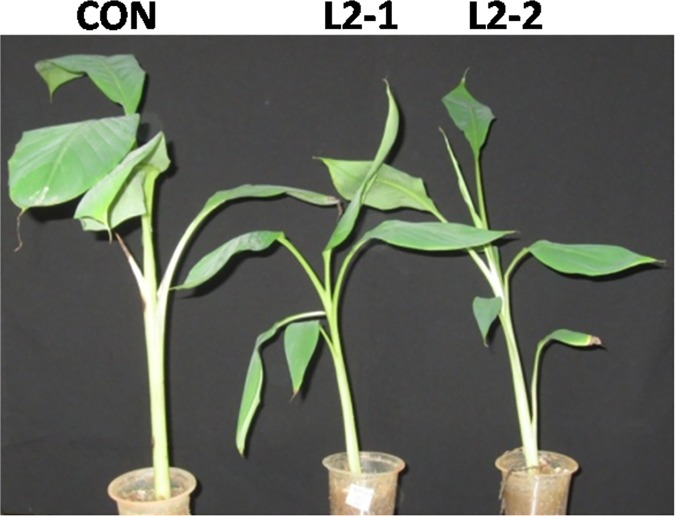
Growth phenotype of transgenic banana overexpressing *MusaMYB31*. Transgenic line (L2) with highest expression of *MusaMYB31* show reduced growth than control plants. The plants were photographed after three months of growth in green house under similar conditions.

Putatively transformed shoots tested for histochemical GUS staining suggested that all the hygromycin resistant lines (L1,L2,L3 and L4)were positive for GUS activity (Fig 5A). Further, the *hpt-II* coding sequence was PCR amplified from genomic DNA of different transgenic lines (Fig 5B). Southern blot analysis performed after isolation of the genomic DNA suggested integration of one to two copies of T-DNA in different transgenic events (Fig 5C). The alteration in expression of *MusaMYB31* in different transgenic lines due to constitutive overexpression was analyzed by quantitative RT-PCR. Fold change in transcript level of *MusaMYB31* in different lines was: 11.2 in line L1, 15.6 in line L2, 5.3 in line L3 and 6.9 in line L4 (Fig 5D). The morphology of transgenic lines and control plants was comparable in early developmental stages suggesting similar morphological development. However, maximum expressing line (L2) after prolonged growth in green house displayed marginal growth stunting relative to control plant (Fig 6).

### Altered secondary wall deposition in transgenic banana

**Fig 7:**
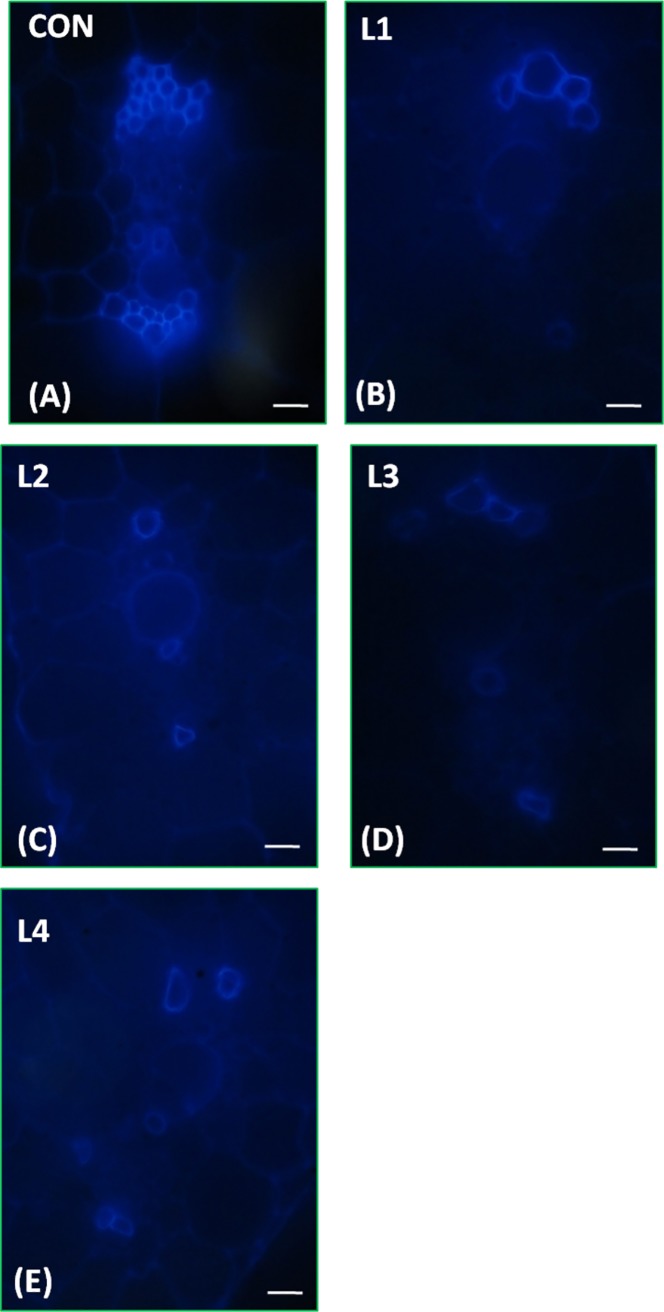
Secondary wall analysis of transgenic lines. Lignin autofluorescence after ultra violet (UV) excitation of petiole sections allowing the visualization of secondary cell deposition around the vascular bundles. Images is taken after focusing on the vascular bundle of petiole of (A) control plant (B-E) transgenic lines L1-L4. Notice the incomplete and non-uniform lignin deposition in transgenic lines. The scale bar corresponds to 10 μm.

**Fig 8:**
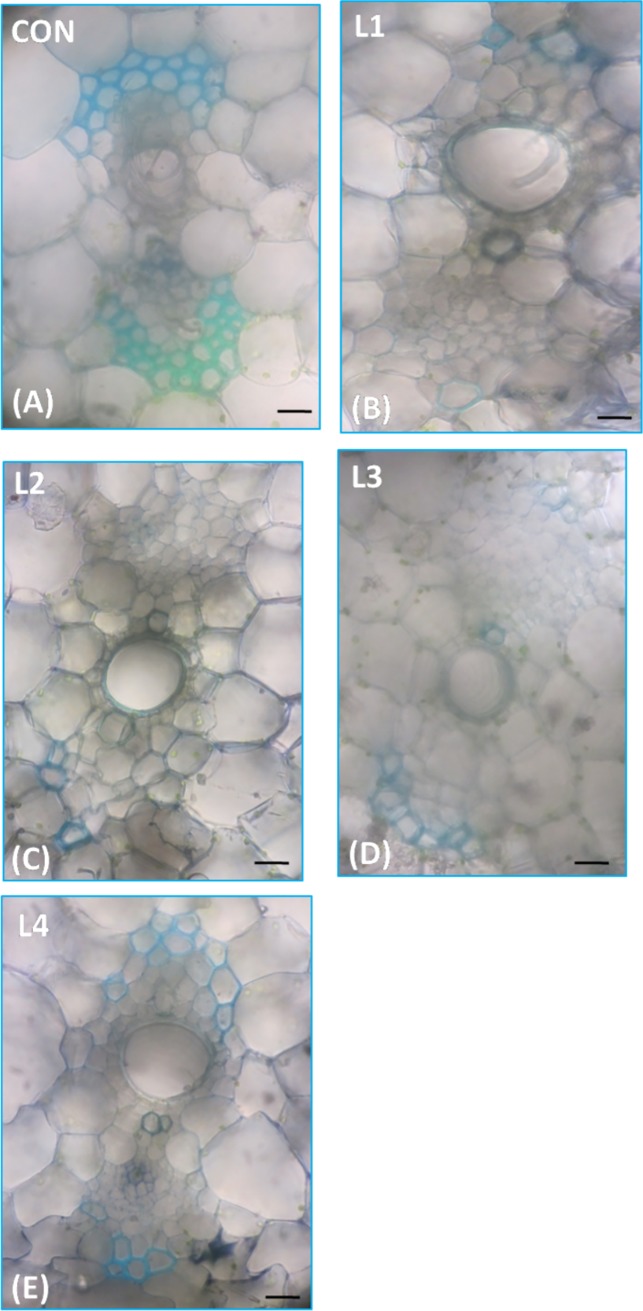
Toluidine blue staining of secondary wall. Toluidine blue stained cross sections of petiole displaying the secondary wall deposition over the vascular tissue. Note the non-uniform and incomplete secondary wall deposition in vascular elements of transgenic lines. Scale bar measures 10 μm.

The lignification in vascular elements of transgenic and control plants were monitored by examination of cross sections under microscope. The secondary wall depositions in vascular elements of banana was visualized by auto fluorescence of lignin under UV (ultra violet) light and by staining the cross section with toluidine blue. Observation of lignin deposition pattern indicated incomplete deposition of lignin in most of the vascular elements of transgenic lines suggesting the repressive activity of MusaMYB31 towards lignin biosynthesis. However, the number of cell layers in vascular elements of control and transgenic lines appeared to be unaltered suggesting MusaMYB31 represses only secondary wall deposition (Fig 7). In majority of transgenic lines, the xylem vessel elements either failed to deposit lignin or stain unsubstantially with toluidine blue (Fig 8).

### Overexpression of *MuaMYB31* represses general phenylpropanoid biosynthesis pathway

**Fig 9:**
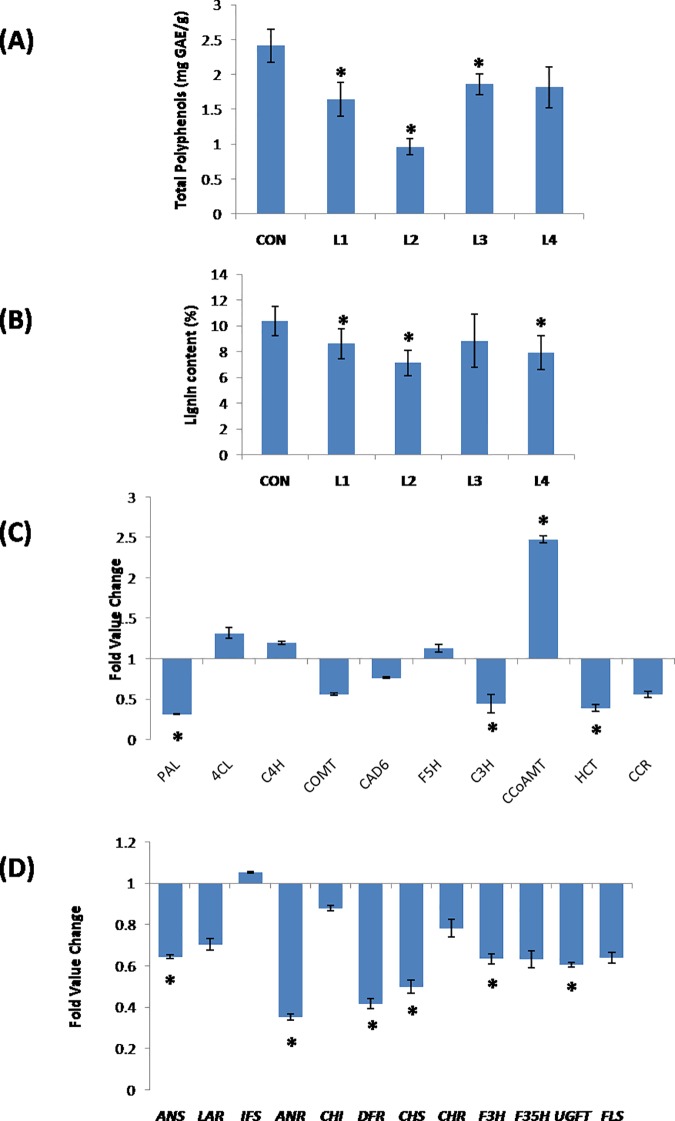
*MusaMYB31* represses general phenylpropanoid biosynthesis pathway. (A) Total polyphenols content of control and transgenic lines. Values represented in terms of mg gallic acid equivalents (GAE)/ gm. (B) Lignin content (%) in cell wall of transgenic lines overexpressing *MusaMYB31* relative to control plant. (C) Overexpression of *MusaMYB31* alters the expression of multiple genes of lignin biosynthesis pathway. (D) Real time PCR analysis of important genes involved in branches of phenylpropanoid biosynthesis pathway. Values represented are mean ± SD. (CON: control; L1,L2,L3 and L4: transgenic lines). Abbreviations are *PAL* (phenylalanine ammonia-lyase), *COMT* (caffeic acid 3-O-methyltransferase), *C3H* (coumarate 3-hydroxylase), *HCT* (hydroxycinnamoyl CoA: shikimate hydroxycinnamoyl transferase), *CCR* (cinnamoyl CoA reductase), *4CL* (4-coumarate: CoA ligase), *C4H* (cinnamic acid 4-hydroxylase), *F5H* (ferulate 5-hydroxylase), *CAD6* (cinnamyl alcohol dehydrogenase 6), *CCoAOMT* (caffeoyl-CoA O-methyltransferase), *ANS* (anthocyanin synthase), *LAR* (leucoanthocyanidin reductase), *ANR* (anthocyanidin reductase), *CHI* (chalcone isomerase), *DFR* (dihydroflavonol 4-reductase), *CHS* (chalcone synthase), *CHR* (chalcone reductase), *F3H* (flavanone 3-hydroxylase), *F35H* (flavonoid 3,5-hydroxylases), *UGFT* (UDP-glucose: flavonoid-3-O-glucosyltransferase), *FLS* (flavonol synthase) and *IFS* (isoflavone synthase). Statistical significant events at 5% are shown with an asterisk (*) on the top.

The effect of *MusaMYB31* overexpression on the secondary wall deposition provided evidence that MusaMYB31 can represses the phenylpropanoid biosynthesis pathway. We have estimated the total polyphenolic content of the control and transgenic lines to support the above observation. Polyphenolic content of all the transgenic lines was remarkably lower than control plants with maximum repression in synthesis of polyphenols observed in transgenic event L2 (Fig 9A). Similarly lignin content was also reduced in the transgenic lines overexpressing *MusaMYB31* supporting the observation of diminished secondary wall depositions in transgenic lines (Fig 9B).The quantum of reduction in polyphenol content as well as lignin in different transgenic lines followed the quantum of overexpression of *MusaMYB31* further proving that the reduction is infact due to overexpression of *MusaMYB31*. Given that MusaMYB31 reduced the deposition of secondary wall in vascular elements and synthesis of polyphenols in transgenic lines, we performed the quantitative RT-PCR analysis of important genes involved in lignin and polyphenols biosynthesis to obtain a broader picture about probable downstream target of MusaMYB31. Total RNA isolated form leaves of transgenic lines and control plants was used for first strand cDNA synthesis, which was subsequently utilized for quantitative RT-PCR analysis. Remarkable reduction in expression of *PAL* (phenylalanine ammonia-lyase), *COMT* (caffeic acid 3-O-methyltransferase), *C3H* (coumarate 3-hydroxylase), *HCT* (hydroxycinnamoyl CoA: shikimate hydroxycinnamoyl transferase) and *CCR* (cinnamoyl CoA reductase) was recorded, while the expression of *4CL* (4-coumarate: CoA ligase), *C4H* (cinnamic acid 4-hydroxylase), *F5H* (ferulate 5-hydroxylase) and *CAD6*(cinnamyl alcohol dehydrogenase 6) appeared to be unaffected by the overexpression of *MusaMYB31* (Fig 9C). The fold changeof different genes observed relative to control expression was: 0.31 fold of *PAL*, 0.56 fold of *COMT*, 0.44 fold of *C3H*, 0.39 fold of *HCT* and0.55 fold of *CCR*. The repression of these important lignin biosynthesis pathway genes is in line with the reduction in the deposition of secondary wall in transgenic lines. However, the expression of *CCoAOMT* (caffeoyl-CoA O-methyltransferase) was elevated due to overexpression of *MusaMYB31* (2.5 fold relative to control expression). Similar elevation in expression of *CCoAOMT* due to overexpression of other R2R3 MYB transcription factors have been reported in other studies as well [11]. We also analyzed other phenyl phenylpropanoid biosynthesis pathway related genes to study the effect of *MusaMYB31* overexpression on their expression. Our analysis suggested that *MusaMYB31* overexpressing transgenic line manifested repression of many of such genes, with fold change in expression relative to control was as follows: 0.64 fold for *ANS* (anthocyanin synthase), 0.7 fold for *LAR* (leucoanthocyanidin reductase), 0.35 fold for *ANR* (anthocyanidin reductase), 0.87 fold of *CHI* (chalcone isomerase), 0.4 fold for *DFR* (dihydroflavonol 4-reductase), 0.49 fold for *CHS* (chalcone synthase), 0.78 fold for *CHR* (chalcone reductase), 0.64 fold for *F3H* (flavanone 3-hydroxylase), 0.63 fold for *F35H* (flavonoid 3,5-hydroxylases), 0.6 fold for *UGFT* (UDP-glucose: flavonoid-3-O-glucosyltransferase) and 0.64 fold for *FLS* (flavonol synthase). The expression of isoflavone synthase (*IFS*) appeared to be unaffected by overexpression of the *MusaMYB31* (Fig 9D). These results indicated that overexpression of *MusaMYB31* has the potential to amend the expression of a whole set of genes involved in phenylpropanoid biosynthesis pathway.

## Discussion

Plants activate production of various primary and secondary metabolites in response to external factors and developmental stages. Transcription factors regulate the time and stage specific production of several of these metabolites. Important among these are members belonging to subgroup4 of R2R3-MYB transcription factors, which have been associated with repression of phenylpropanoid pathway including lignin and polyphenols biosynthesis [12, 10, 15]. In this report we have characterized the first R2R3 MYB transcription factor associated with repression of general phenylpropanoid biosynthesis pathway in an economically important fruit crop, banana. MusaMYB31 contain all the important motifs like C1 (llsrGIDPxTHR), C2 (pdLNL[D/E]L), C3/ZnF like and C4 motifs which are characteristic of subgroup 4 of R2R3 MYB transcription factors [7]. The ethylene response factor-associated amphiphilic repression (EAR) motif (LxLxL pattern) of MusaMYB31 has sequence conservation with EAR motif (DLNxxP or LxLxL) of AtMYB4, AtMYB7 and AtMYB32 [26, 27], suggesting the repression activity of MusaMYB31 towards transcription of other genes [7, 25]. C- terminal end of MusaMYB31 also contain C4 motif which has been detected in some repressors of phenylpropanoid pathway and lignin biosynthesis pathway, like maize ZmMYB31 and ZmMYB42 [2, 13], petunia PhMYB4 [28] and switchgrass PvMYB4 (conserved sequence of C4 motif: FLGLX_4–7_V⁄LLD⁄GF⁄YR⁄SX1LEMK) [15].

We successfully regenerated transgenic banana plants overexpressing MusaMYB31 by *Agrobacterium* mediated transformation of embryogenic cell suspensions of *Musa* cultivar Rasthali, which is an economically important banana crop. Four transgenic lines were regenerated and were confirmed for transgene integration by GUS staining, PCR analysis and Southern blot analysis. PCR analysis for T-DNA integration was conducted by analyzing the amplification of *hpt-II* from the genomic DNA as *hpt-II* coding sequence is present within the T-DNA border sequences. Southern blot indicated that all the transgenic lines were originated from independent transformation events as number of T-DNA copies and their position on autoradiograph were variable among different lines. It was difficult to correlate the T-DNA copy number and *MusaMYB31* overexpression in different lines, possibly due to factor of differential locus integration of T-DNA (position effect). Transgenic lines with high expression of *MusaMYB31* displayed marginal growth retardation relative to control plants at later stages of growth in the green house. Similar, growth retardation due to overexpression of R2R3 MYB repressors has been well documented [11]. Other reported morphological alteration observed in transgenic plants overexpressing R2R3 MYB repressors such as changed leaf curvature and white lesions on older leaves was not observed in the present study. Similar growth retardation without appearance of white lesions have also been reported in case of transgenic plants overexpressing *LlMYB1*, a R2R3 MYB transcription factor from *Leucaena leucocephala* [11].

Overexpression of *MusaMYB31* could down regulate many genes of general phenylpropanoid as well as lignin biosynthesis pathway. Strong repression of *PAL*, *C3H* and *HCT* was observed in transgenic lines while expression of *CCoAOMT* was marginally elevated suggesting probable differential regulation of these genes by *MusaMYB31*.A few reports on regulation of lignin deposition and phenylpropanoid pathway in fruit crops, specially loquat, pear, grape and apple has emerged in recent years[29–32]. In Loquat fruit, *EjMYB8* and *EjMYB9* were induced in response to chilling injury causing elevated lignin content, while heat treatment inhibit their expression reducing lignification [29]. *EjMYB8* regulate *Ej4CL1* promoter and transient overexpression of *EjMYB8* in tobacco and loqaut leaves increased lignin content suggesting *EjMYB8* is a functional regulator of lignin deposition [29]. Majority of the work on regulation of lignin biosynthesis pathway has been focused on members of *NAC* and *MYB* transcription factor family. Recently the involvement of *AP2/ERF* family members in regulation of lignin deposition has been shown. *Arabidopsis* AP2/ERF factor, SHINE can bind to the promoters of *MYB 58* and *SND1* among others [33]. *Eriobotrya japonica* AP2 transcription factor, EjAP2-1 has been shown to indirectly repress chilling induced lignin biosynthesis in fruit and the repression has been attributed to EAR motifs and its interaction via lignin biosynthesis-related EjMYB1 and EjMYB2 [34]. Another MYB transcription factor EjODO1, in Loquat (*Eriobotrya japonica*) fruit lignification has been recently reported. Expression of *EjODO1* decreased with reduction in lignification and its transient overexpression elevated lignin content [35]. Recent annotation of *MYB* genes in Chinese pear (*Pyrus bretschneideri Rehd*.)identified 129 *MYB* genes and expression of *PbMYB25* and *PbMYB52* during pear fruit development indicated their role in regulation of lignin biosynthesis [30]. The fruit pericarp hardening of mangosteen (*Garcinia mangostana L*.) is attributed to activation of lignin biosynthesis pathway and an *R2R3 MYB* transcription factor, *GmMYB30*[36]. Maize transcription factors, ZmMYB31 and ZmMYB42 which show high sequence identity with MusaMYB31 are also reported to repress lignin biosynthesis pathway genes including *PAL*, *COMT*, *HCT*, *CAD6*,*C4H* and *4CL*[2, 13]. Reduction in total polyphenols content and lignin was observed in all the transgenic lines which was in line with repression of many genes involved in flavonoids, lignin and polyphenols biosynthesis. Two MYB transcription factors, EjMYB1 and EjMYB2 act as transcriptional activator and repressor respectively in regulation of Loquat (*Eriobotrya japonica*) fruit lignification as they regulate the promoter of lignin biosynthesis genes [37].Strong repression of important genes like *ANS*, *ANR*, *DFR*, *F3H* among others in transgenic lines suggest downregulation due to overexpression of *MusaMYB31*. Maize ZmMYB42 can also reduce total polyphenols content and repress *F3H* and *F3’H* expression [2]. Anthocyanins are vacuolar pigments and their biosynthesis is through one of the branches of general phenylpropanoid pathway [38]. Some of the recent studies on regulation of anthocyanin in fruit crops by MYB transcription factors have been carried out. In apple (*Malus domestica*),anthocyanin biosynthesis is regulated by a *MYB* transcription factor, *MdMYB10*, and it’s over expression induced accumulation of anthocyanin patches [32]. Expression of *PyMYB10*, from Asian pear (*Pyrus pyrifolia* cv. ‘*Aoguan*)is related to anthocyanin accumulation in fruit and its overexpression induced pigmentation in immature seeds of transgenic *Arabidopsis*[39]. *MYB* transcription factor (*VlmybA1-1*) in grape regulating anthocyanin biosynthesis via expression of *UDP-glucose*: *flavonoid 3-O-glucosyltransferase* (*UFGT*) was isolated and its transient overexpression in grape somatic embryos induced ectopic anthocyanin deposition [31]. The reduction in lignin deposition in vascular elements of transgenic lines overexpressing *MusaMYB31* suggested the potential application of this transcription factor in genetic improvement of banana for its utilization as a biofuel crop. Recent studies have shed some light on the genetic regulation of lignin deposition in banana [21, 22]. Present study has provided an alternative approach for genetic improvement of banana in the context of biofuel production.

## Supporting information

S1 Table Sequences of various primer pairs used in the study. (DOCX)

## References

[1] Boerjan W, Ralph J, Baucher M (2003) Lignin biosynthesis. Annu Rev Plant Biol 54:519–546 10.1146/annurev.arplant.54.031902.134938 14503002

[2] Sonbol FM, Fornalé S, Capellades M, Encina A, Touriño S, Torres JL, Rovira P, Ruel K, Puigdomènech P, Rigau J, Caparrós-Ruiz D (2009) The maize ZmMYB42 represses the phenylpropanoid pathway and affects the cell wall structure, composition and degradability in *Arabidopsisthaliana*. Plant Mol Biol.70:283–96 10.1007/s11103-009-9473-2 19238561

[3] Cai Y, Zhang K, Kim H, Hou G, Zhang X, Yang H, Feng H, Miller L, Ralph J, Liu CJ (2016) Enhancing digestibility and ethanol yield of Populus wood via expression of an engineered monolignol 4-O-methyltransferase. Nat Commun. 10.1038/ncomms11989 PMC4931242 27349324

[4] Torney F, Moeller L, Scarpa A, Wang K (2007) Genetic engineering approaches to improve bioethanol production from maize. Curr Opin Biotechnol 18:193–199. 10.1016/j.copbio.2007.03.006 17399975

[5] Vanholme R, Morreel K, Ralph J, Boerjan W (2008) Lignin engineering. Curr Opin Plant Biol 11:1–8 10.1016/j.pbi.2008.03.005 18434238

[6] Zhong R, Lee C, Ye ZH. (2010) Evolutionary conservation of the transcriptional network regulating secondary cell wall biosynthesis. Trends in Plant Science 15:625–632 10.1016/j.tplants.2010.08.007 20833576

[7] Kranz HD, Denekamp M, Greco R, Jin H, Leyva A, Meissner RC, Petroni K, Urzainqui A, Bevan M, Martin C, et al (1998) Towards functional characterisation of the members of the R2R3-MYB gene family from *Arabidopsisthaliana*. Plant J 16: 263–276 9839469 10.1046/j.1365-313x.1998.00278.x

[8] Legay S, Lacombe E, Goicoechea M, Briere C, Se´guin A, Mackay J, Grima-Pettenati J. (2007) Molecular characterization of *EgMYB1*, a putative transcriptional repressor of the lignin biosynthetic pathway. Plant Science 173:542–549.

[9] Zhao Q, Dixon RA (2011) Transcriptional networks for lignin biosynthesis: more complex than we thought? Trends Plant Sci 16:227–233 10.1016/j.tplants.2010.12.005 21227733

[10] Jin H, Cominelli E, Bailey P, Parr A, Mehrtens F, Jones J, Tonelli C, Weisshaar B, Martin C (2000) Transcriptional repression by *AtMYB4* controls production of UV-protecting sunscreens in *Arabidopsis*. EMBO J 19: 6150–6161 10.1093/emboj/19.22.6150 11080161 PMC305818

[11] Omer S, Kumar S, Khan BM (2013) Over-expression of a subgroup 4 R2R3 type MYB transcription factor gene from *Leucaena leucocephala* reduces lignin content in transgenic tobacco. Plant Cell Rep.32:161–71 10.1007/s00299-012-1350-9 23052594

[12] Tamagnone L, Merida A, Parr A et al (1998) The AmMYB308 and AmMYB330 transcription factors from *Antirrhinum* regulate phenylpropanoid and lignin biosynthesis in transgenic tobacco. Plant Cell 10:135–154 9490739 10.1105/tpc.10.2.135 PMC143979

[13] Fornalé S, Shi X, Chai C, Encina A, Irar S, Capellades M, Fuguet E, Torres JL, Rovira P, Puigdomènech P et al (2010) ZmMYB31 directly represses maize lignin genes and redirects the phenylpropanoid metabolic flux. Plant J 64: 633–644 10.1111/j.1365-313X.2010.04363.x 21070416

[14] Cavallini E, Matus JT, Finezzo L, Zenoni S, Loyola R, Guzzo F, Schlechter R, Ageorges A, Arce-Johnson P, Tornielli GB (2015) The phenylpropanoid pathway is controlled at different branches by a set of R2R3-MYB C2 repressors in grapevine. Plant Physiol.167:1448–70 10.1104/pp.114.256172 25659381 PMC4378173

[15] Shen H, He X, Poovaiah CR, Wuddineh WA, Ma J, Mann DG, Wang H, Jackson L, Tang Y, Stewart CN Jr, Chen F, Dixon RA (2012) Functional characterization of the switchgrass (Panicum virgatum) R2R3-MYB transcription factor PvMYB4 for improvement of lignocellulosic feedstocks. New Phytol.193:121–36 10.1111/j.1469-8137.2011.03922.x 21988539

[16] Schmittgen TD, Livak KJ (2008) Analyzing real-time PCR data by the comparative C(T) method. Nat Protoc. 3:1101–8 18546601 10.1038/nprot.2008.73

[17] Kumar S, Nei M, Dudley J, Tamura K (2008) MEGA: biologist-centric software for evolutionary analysis of DNA and protein sequences. Briefings in bioinformatics 9:299–306z 10.1093/bib/bbn017 18417537 PMC2562624

[18] Ganapathi TR, Higgs NS, Balint Kurti PJ, Arntzen CJ, May GD, Van Eck JM (2001) *Agrobacterium* mediated transformation of embryogenic cell suspensions of the banana cultivar *Rasthali* (AAB). Plant Cell Rep. 20:157–162 10.1007/s002990000287 30759903

[19] Hood EE, Gelvin SB, Melchers LS, Hoekama A (1993) New *Agrobacterium* helper plasmids for gene transfer to plants. Transgenic Res 2:208–218

[20] Cote FX, Domergue R, Monmarson S, Schwendiman J, Teisson C, Escalant JV (1996) Embryogenic cell suspensions from the male flower of *Musa* AAA cv. Grand Nain. Physiol Plant. 97:285–290

[21] Negi S, Tak H, Ganapathi TR (2015) Cloning and functional characterization of *MusaVND1* using transgenic banana plants. Transgenic Res. 24:571–85 10.1007/s11248-014-9860-6 25523085

[22] Negi S, Tak H, Ganapathi TR (2015) Functional characterization of secondary wall deposition regulating transcription factors *MusaVND2* and *MusaVND3* in transgenic banana plants. Protoplasma10.1007/s00709-015-0822-5 25952082

[23] Ogata K, Kanei-Ishii C, Sasaki M et al (1996) The cavity in the hydrophobic core of Myb DNA-binding domain is reserved for DNA recognition and trans-activation. Nat Struct Biol 3:178–18 8564545 10.1038/nsb0296-178

[24] Zimmermann IM, Heim MA, Weisshaar B, Uhrig JF (2004) Comprehensive identification of *Arabidopsis thaliana* MYB transcription factors interacting with R/B-like BHLH proteins. Plant J 40:22–34 10.1111/j.1365-313X.2004.02183.x 15361138

[25] Hiratsu K, Matsui K, Koyama T, Ohme-Takagi M (2003) Dominant repression of target genes by chimeric repressors that include the EAR motif, a repression domain, in *Arabidopsis*. Plant J 34: 733–739 12787253 10.1046/j.1365-313x.2003.01759.x

[26] Kagale S, Links MG, Rozwadowski K (2010) Genome-wide analysis of ethylene-responsive element binding factor-associated amphiphilic repression motif-containing transcriptional regulators in *Arabidopsis*. Plant Physiol 152: 1109–1134 10.1104/pp.109.151704 20097792 PMC2832246

[27] Stracke R, Werber M, Weisshaar B (2001) The R2R3-MYB gene family in *Arabidopsis thaliana*. Curr Opin Plant Biol 4: 447–456 11597504 10.1016/s1369-5266(00)00199-0

[28] Colquhoun TA, Kim JY, Wedde AE, Levin LA, Schmitt KC, Schuurink RC, Clark DG (2011) *PhMYB4* fine-tunes the floral volatile signature of *Petunia* x *hybrida* through *PhC4H*. J Exp Bot 62: 1133–1143 10.1093/jxb/erq342 21068208 PMC3022401

[29] Wang WQ, Zhang J, Ge H, Li SJ, Li X, Yin XR, Grierson D, Chen KS (2016) *EjMYB8* Transcriptionally Regulates Flesh Lignification in Loquat Fruit. PLoS One.11:e0154399 10.1371/journal.pone.0154399 27111303 PMC4844104

[30] Cao Y, Han Y, Li D, Lin Y, Cai Y (2016) MYB Transcription Factors in Chinese Pear (Pyrus bretschneideri Rehd.): Genome-Wide Identification, Classification, and Expression Profiling during Fruit Development. Front Plant Sci. 7:577 10.3389/fpls.2016.00577 27200050 PMC4850919

[31] Kobayashi S, Ishimaru M, Hiraoka K, Honda C (2002) *Myb*-related genes of the Kyoho grape (*Vitis labruscana*) regulate Anthocyanin biosynthesis. Planta 215:924–933 10.1007/s00425-002-0830-5 12355152

[32] Espley RV, Hellens RP, Putterill J, Stevenson DE, Kutty-Amma S, Allan AC (2007) Red colouration in apple fruit is due to the activity of the *MYB* transcription factor, *MdMYB10*. Plant J. 49:414–27 10.1111/j.1365-313X.2006.02964.x 17181777 PMC1865000

[33] Ambavaram MM, Krishnan A, Trijatmiko KR, Pereira A (2011) Coordinated activation of cellulose and repression of lignin biosynthesis pathways in rice. Plant Physiol. 155:916–31 10.1104/pp.110.168641 21205614 PMC3032476

[34] Zeng JK, Li X, Xu Q, Chen JY, Yin XR, Ferguson IB, Chen KS (2015) *EjAP2-1*, an AP2/ERF gene, is a novel regulator of fruit lignification induced by chilling injury, via interaction with *EjMYB* transcription factors. Plant Biotechnol J.13:1325–34 10.1111/pbi.12351 25778106

[35] Zhang J, Ge H, Zang C, Li X, Grierson D, Chen KS, Yin XR (2016) *EjODO1*, a *MYB* Transcription Factor, Regulating Lignin Biosynthesis in Developing Loquat (*Eriobotrya japonica*) Fruit. Front Plant Sci.7:1360 10.3389/fpls.2016.01360 27695460 PMC5025436

[36] Kamdee C, Imsabai W, Kirk R, Allan AC, Ferguson IB, Ketsa S (2014) Regulation of lignin biosynthesis in fruit pericarp hardening of mangosteen (*Garcinia mangostana* L.) after impact. Postharvest Biol. Technol. 97: 68–76.

[37] Xu Q, Yin XR, Zeng JK, Ge H, Song M, Xu CJ, Li X, Ferguson IB, Chen KS (2014) Activator- and repressor-type *MYB* transcription factors are involved in chilling injury induced flesh lignification in loquat via their interactions with the phenylpropanoid pathway. J Exp Bot. 65:4349–59 10.1093/jxb/eru208 24860186 PMC4112638

[38] Shi MZ, Xie DY (2014) Biosynthesis and metabolic engineering of anthocyanins in *Arabidopsis thaliana*. Recent Pat Biotechnol. 8:47–60 10.2174/1872208307666131218123538 24354533 PMC4036305

[39] Feng S, Wang Y, Yang S, Xu Y, Chen X (2010) Anthocyanin biosynthesis in pears is regulated by a *R2R3-MYB* transcription factor *PyMYB10*. Planta. 232:245–55 10.1007/s00425-010-1170-5 20422209

