# Supplementary material for: Overexpression of MusaMYB31, a R2R3 type MYB transcription factor gene indicate its role as a negative regulator of lignin biosynthesis in banana
Source: PLoS One. 2017 Feb 24;12(2):e0172695. doi: 10.1371/journal.pone.0172695 (PMC5325293; doi:10.1371/journal.pone.0172695)
Supplement: S1 Table — (DOCX) [file pone.0172695.s001.docx]

**Supplemental table**

**Primers for cloning and other molecular work done in the study**

|  |  | **Forward Primer (5’ - 3’)** | **Reverse Primer (5’ -3’)** |
| --- | --- | --- | --- |
| 1 | **Primers for cloning *MusaMYB31*  in pCAMBIA1301** | aCctgcaggATGGGGAGATCGCCATGC | tggtaccTTACTTCATCTTCAGGCCTCTGTAGC |
| 2 | ***MusaMYB31*  real time PCR primers** | AACCTGGAGCTGTGCATAAG | TCAGGCCTCTGTAGCTCAAC |
| 3 | **Hygromycin phosphotransferase (*hpt)* gene** | GTCCTGCGGGTAAATAGCTG | ATTTGTGTACGCCCGACAGT |
| 4. | ***Musa EF1α* real time PCR primers** | CCGATTGTGCTGTCCTCATT | TTGGCACGAAAGGAATCTTCT |

**Lignin biosynthesis pathway genes and primers**

| **Gene** | **Banana genome locus identifier** | **Forward Primer (5’ to 3’)** | **Reverse Primer (5’ to 3’)** |
| --- | --- | --- | --- |
| ***PAL*** | GSMUA_Achr5T18560_001 | CTTGGACTACGGCTTCAAGG | CTTGAGGTTCTCCTCCAGGT |
| ***COMT*** | GSMUA_Achr3T27470_001 | CATGATCACCTCCAAACACC | AACGCACTCCACCACTATCA |
| ***4CL*** | GSMUA_Achr1T23450_001 | GCGACAAGGAACACCATAGA | CATTCGACCTCACGACAAAC |
| ***HCT*** | GSMUA_Achr6T01940_001 | CAGAAGGGCTTATGGGTGAT | TCAGAGCCTCGAGTTCCTC |
| ***CAD6*** | GSMUA_Achr5T11490_001 | CGCTGATGATTTCCTCGTTA | CTCCTGCGTCTCCTTCATC |
| ***C4H*** | GSMUA_Achr6T08670_001 | AGCAAGATCCTGGTCAACG | GTATGTGGAGGCTGAACTGG |
| ***CCoAMT*** | GSMUA_Achr6T36400_001 | GCTCCTCAAACTGATCAACG | CGACCCATGGTTCTTCTCAT |
| ***CCR*** | GSMUA_Achr5T04460_001 | GGTGTCTTCCACACTGCTTC | CCTTCCCGTAGCAATACCAG |
| ***C3H*** | GSMUA_Achr4T10110_001 | TCAGGGAGGACGAGGTAATC | CAGAGAATTCCGATGATGGAG |
| ***F5H*** | GSMUA_Achr11T17500_001 | CAAATCAGCATGGGAGGAC | GAATTAGATGAGAGGAGGTGCAG |

**Genes of phenyl propanoid biosynthesis pathway and their real time primers**

|  | **Name** | **Banana genome locus identifier** | **Forward Primer (5’ - 3’)** | **Reverse Primer (5’ -3’)** |
| --- | --- | --- | --- | --- |
| **1** | **CHS** | GSMUA_Achr6T08180_001 | ATCTTCTGGATCGCGCAC | ACGTGGGAAGAAATGGCG |
| **2** | **CHI** | GSMUA_Achr4T16830_001 | TGGAGCAGTTCAAAGAGGCT | AGCTTGGTCTCTTCTGCCTC |
| **3** | **F3’H** | GSMUA_Achr8T05810_001 | TGAACGGCTACTACATCCCC | TTCTCCTCCATGTCGAGCTC |
| **4** | **F3’5’H** | GSMUA_Achr2T00210_001 | CACACGGCTCATAGTCAACG | TCCTTCATGTCGAGCTCCTC |
| **5** | **DFR** | GSMUA_Achr4T24540_001 | CTTGTTCGAGCATCCGGAAG | TCGACCTGCTCTTTCTTGGT |
| **6** | **ANS** | GSMUA_Achr5T04080_001 | GGACTCCATCATCATGCACG | CCCGAGTCTTCTTGAAGAGC |
| **7** | **FLS** | GSMUA_Achr10T25240_001 | TCCCTAACCTTGTGCCGG | AGTCTTTGTACTTCTTCGGCTT |
| **8** | **UGFT** | GSMUA_Achr10T23660_001 | GCCGCAGTTGGACGTC | CGCGTCGTCATGGCCT |
| **9** | **ANR** | GSMUA_Achr8T01490_001 | CTTCCGGCCGCTACATCT | TGCTCCCTTCCCAGTTGT |
| **10** | **IFS** | GSMUA_Achr8T25390_001 | GGGATTTTCTGAAGCGGAGG | TCCATGCAGCTTCCACCC |
| **11** | **LAR** | GSMUA_AchrUn_randomT18370_001 | GAGGACCTGCTCGCCATT | TGATCCACCGTAGCAGCG |
| **12** | **CHR** | GSMUA_Achr7T12390_001 | GGGTTCGACTGGGACTCC | CCAGTGACTTGTAAGGGCC |
